# BioMAT: An Open-Source Biomechanics Multi-Activity Transformer for Joint Kinematic Predictions Using Wearable Sensors

**DOI:** 10.3390/s23135778

**Published:** 2023-06-21

**Authors:** Mohsen Sharifi-Renani, Mohammad H. Mahoor, Chadd W. Clary

**Affiliations:** 1 Center for Orthopaedic Biomechanics, University of Denver, Denver, CO 80208, USA; 2 Computer Vision and Social Robotics Laboratory, University of Denver, Denver, CO 80208, USA

**Keywords:** deep learning, transformer, joint kinematics, wearable, IMUs, stair ascent, stair descent, gait

## Abstract

Through wearable sensors and deep learning techniques, biomechanical analysis can reach beyond the lab for clinical and sporting applications. Transformers, a class of recent deep learning models, have become widely used in state-of-the-art artificial intelligence research due to their superior performance in various natural language processing and computer vision tasks. The performance of transformer models has not yet been investigated in biomechanics applications. In this study, we introduce a Biomechanical Multi-activity Transformer-based model, BioMAT, for the estimation of joint kinematics from streaming signals of multiple inertia measurement units (IMUs) using a publicly available dataset. This dataset includes IMU signals and the corresponding sagittal plane kinematics of the hip, knee, and ankle joints during multiple activities of daily living. We evaluated the model’s performance and generalizability and compared it against a convolutional neural network long short-term model, a bidirectional long short-term model, and multi-linear regression across different ambulation tasks including level ground walking (LW), ramp ascent (RA), ramp descent (RD), stair ascent (SA), and stair descent (SD). To investigate the effect of different activity datasets on prediction accuracy, we compared the performance of a universal model trained on all activities against task-specific models trained on individual tasks. When the models were tested on three unseen subjects’ data, BioMAT outperformed the benchmark models with an average root mean square error (RMSE) of 5.5 ± 0.5°, and normalized RMSE of 6.8 ± 0.3° across all three joints and all activities. A unified BioMAT model demonstrated superior performance compared to individual task-specific models across four of five activities. The RMSE values from the universal model for LW, RA, RD, SA, and SD activities were 5.0 ± 1.5°, 6.2 ± 1.1°, 5.8 ± 1.1°, 5.3 ± 1.6°, and 5.2 ± 0.7° while these values for task-specific models were, 5.3 ± 2.1°, 6.7 ± 2.0°, 6.9 ± 2.2°, 4.9 ± 1.4°, and 5.6 ± 1.3°, respectively. Overall, BioMAT accurately estimated joint kinematics relative to previous machine learning algorithms across different activities directly from the sequence of IMUs signals instead of time-normalized gait cycle data.

## 1. Introduction

Accurate measurement and prediction of joint kinematics enable the development of tools for pathological diagnosis, implant design, rehabilitation, sports science, and ergonomics [1,2,3,4,5]. Passive-marker motion capture (MOCAP) systems are the current gold standard in measuring joint kinematics. However, the use of these systems is time-consuming, restricted to lab environments, and requires technical expertise [6,7,8,9]. In contrast, wearable inertial measurement units (IMUs) have gained attention in biomechanics applications and joint kinematic measurement due to their portability, ease of use, and low cost. 

Deep learning, a subset of machine learning, has significantly advanced the capability to convert IMU signals into joint kinematics. Among those, Mundt et al. tested various deep neural network (NN) models, including multi-layer perceptron, convolutional neural network (CNN), and recurrent neural networks (RNNs) such as long short-term memory (LSTM) models, in their ability to estimate joint kinematics and kinetics from measured IMU signals during gait [10,11]. Mundt et al. extended their training dataset to include both simulated and measured IMU data to estimate joint kinematic profiles using artificial NNs [12]. Dorschky et al. also found that the addition of synthetic IMU data improved their model predictions [13]. McCabe et al. incorporated a force-measuring insole with an IMU on the shank to predict hip joint loading along with kinematics using a neural network model [14]. However, these studies were restricted to walking and treadmill activities. Recently, Tan et al. implemented a Bidirectional LSTM (BiLSTM) model to estimate joint kinematics in the sagittal plane using IMUs for osteoarthritis (OA) patients performing activities of daily living: gait, sit-to-stand, and negotiating stairs. Hossain et al. achieved a low error rate in lower extremity joint kinematic predictions using feet IMUs across level walking, treadmill, ramp, stair ascent, and stair descent activities with DeepBBWAE-Net, an ensemble CNN-RNN based deep learning model [15].

A common preprocessing step for deep learning model development is segmentation of kinematics data and the corresponding IMUs signals into individual gait cycles with a consistent length achieved by normalizing the data with respect to time [12,13,15,16,17,18]. Most previous studies used MOCAP data for segmentation. In a novel approach, Mundt et al. predicted joint kinematics based on a continuous stream of IMU data without prior segmentation [16]. They then compared the performance of the LSTM model trained on a longer motion sequence against time-normalized gait cycles and found that a longer motion sequence resulted in superior performance. Hernandez et al. also utilized a continuous time series for training their convolutional neural network long short-term memory model (CNNLSTM) but studied its performance in other activities such as walking, running, and gait transition [17]. 

In practical applications where subjects wear only IMUs, segmentation of cycles with kinematic data is not feasible. One possible solution is segmentation based on the characteristics of the IMU data. Proposed methods to segment IMU data are currently limited to gait activities and healthy populations [17]. The feasibility of these methods for applications in complex activities of daily living, such as transitioning from gait to stair ascent, turning, sitting to walking, etc., has not been fully investigated. These methods may not apply to individuals with musculoskeletal pathology as they produce abnormal movement patterns [9,19,20,21,22,23,24]. The time and computational cost of the additional preprocessing steps required for continuous real-time joint kinematic estimation reduce the desirability of this approach.

While progress has been made, advancements in machine learning methods in biomechanics remain comparably slow to similar applications in language processing and image recognition. One impeding factor is the lack of publicly available datasets, source codes, and models. This limits the development and evaluation of models to only a small group of researchers and delays progress. Publicly available models would allow for additional opportunities to implement state-of-the-art machine learning techniques such as transfer learning [22,23], fine-tuning, or one-shot and zero-shot learning. Thanks to Camargo et al., a publicly available dataset has been introduced containing 3-dimensional kinematics and wearable sensor data from 22 adults for multiple locomotion tasks including level walking (LW), ramp ascent (RA), ramp descent (RD), stair ascent (SA), and ramp descent (SD) [25]. Using such datasets, machine learning models for various applications can be developed and there is a greater opportunity for researchers to advance the field [26]. As of this time, there are no publicly available kinematic-prediction models.

Machine learning models that have been used in previous studies were limited to NN models, including RNN, CNN, LSTM, and fully connected NNs in various combinations [13,15,16,17,18,27,28,29]. These models provided reliable performance in mapping IMU signals to joint kinematics. However, recent research in the field of deep learning has shown that a relatively new model, the transformer, outperformed previous models in many tasks and is increasingly the model of choice for solving deep learning problems. The transformer was introduced in 2017 by a team at Google Brain for natural language processing tasks to overcome the limitations of RNNs for sequence data [30]. RNNs have difficulty capturing long-term dependencies and processing sequential data in parallel. A transformer, on the other hand, uses self-attention to capture global dependencies while processing sequences in parallel. Transformers have evolved beyond language tasks into other areas such as time series analysis [31,32,33,34] and computer vision [35,36]. The potential of this model in biomechanics tasks has not yet been investigated.

We propose the use of transformer models in biomechanics applications to address the current limitations of traditional machine learning models, including the requirements of segmenting gait cycles and the restricted number activities for which a single model can effectively predict lower limb kinematics. The current study has three aims. The first aim is to implement transformer-based models for predicting joint kinematics from continuous streams of unsegmented IMU signals across gait, ramp, and stair activities. The second aim is to compare the performance of transformer-based models against previously published models such as BiLSTM and CNNLSTM. The final aim is to investigate whether a single universal model for all activities has superior performance compared to activity-specific models. We hypothesize that (1) the transformer-based model will outperform previous models in predicting joint kinematics and (2) activity-specific models will perform equivalent to models trained across all activities. These hypotheses will be evaluated by comparing root mean square errors (RMSE) in kinematic predictions between (1) a transformer-based model and previously published machine learning architectures and (2) transformer-based models trained on single-activity datasets and multi-activity datasets. The trained models from this work will be open source, enabling studies of reproducibility and the advancement of the field.

## 2. Materials and Methods

### 2.1. Dataset

A publicly available lower limb biomechanics dataset has been used in this study [25]. This comprehensive dataset includes IMU data along with the kinematic and kinetic profiles of joint biomechanics from 19 healthy subjects (11 male, 8 female, age = 20.5 ± 1.2 years old, height = 1.7 ± 0.1 m, and weight = 68.4 ± 11.7 kg) performing LW, RA, RD, SA, and SD. Each subject was outfitted unilaterally on the right side with 4 six-axis IMUs (3-Space^TM^, Yost Labs, Yost, OH, USA), and bilaterally with 32 motion capture markers (Vicon. Ltd., Oxford, UK). IMUs were attached to the anterior surface of the foot, shank, and thigh at ¾ of the length of each segment and the anterior surface of the torso between the sternum and navel. Ground reaction forces were also recorded using force plates (Bertec, OH, USA) located in the instrumented treadmill and level with the floor, ramp, and stairs. Joint kinematics and kinetics were calculated by analyzing the MOCAP data along with ground reaction forces using inverse kinematics and inverse dynamics in OpenSim [25,37]. The current study utilized data from the IMUs on the lower limb (foot, shank, and thigh) and sagittal plane joint kinematics at the hip, knee, and ankle from the MOCAP for 19 subjects across five activities, including LW at three self-selected speeds, RA, RD, SA, and SD. The cumulative number of activity trials performed by the cohort of subjects prior to segmentation was 1170, 1204, 1204, 789, and 789, for LW, RA, RD, SA, and SD, respectively.

### 2.2. Preprocessing

IMU and kinematic data were down-sampled from 200 Hz to 100 Hz to reduce the overall size of the training set. The time series data were segmented into samples of 256 contiguous points using a sliding window with 50% overlap between subsequent samples. The starting point of each sample was chosen randomly so as not to intentionally coincide with a particular gait event (e.g., heel strike). A zero-padding technique was used to ensure the data were a consistent length prior to use in the deep-learning models. The length of 256 was selected to ensure that each sample consisted of at least two successive gait cycles and limitations of the graphic processing units (GPUs) used during training and evaluation. The IMU data were scaled using the standardization method to facilitate gradient descent convergence during training [26]. A total of 2523, 3369, 3491, 1451, and 1258 samples were generated for LW, RA, RD, SA, and SD, respectively.

### 2.3. Neural Network Models

Three conventional deep NN models (BiLSTM, CNNLSTM, and a new biomechanical multi-activity transformer-based model called BioMAT) and a multi-linear regression (MLR) model were used for mapping IMU data to the sagittal plane kinematics of the hip, knee, and ankle.

#### 2.3.1. Multi-Linear Regression Model

A MLR model was chosen as the baseline for this study as this is a fundamental technique in machine learning and is widely used due to its simplicity and interpretability. Therefore it serves as a baseline for comparisons with more complex models. Input data to the MLR were a matrix where each row contained the concatenated IMU signals for a particular sample. The MLR model included coefficients that were optimized by minimizing the residual sum of squares between the measured and predicted joint kinematics.

#### 2.3.2. CNNLSTM Architecture

CNNLSTM is an architecture specifically designed for sequence prediction with spatial inputs such as images or videos. CNNLSTMs consist of multiple convolutional layers, followed by multiple LSTM layers and a final dense or fully connected layer [17,38,39,40]. Feature extraction occurs with convolutional layers (spatial domain) while time-series prediction is accomplished with recurrent layers (time domain). This model has been used for activity recognition and joint kinematic predictions in previous studies [17,38,39,40]. The current study implemented a Deep CNNLSTM based on Hernandez et al. [17] with two 2D CNN layers followed by two LSTM layers.

#### 2.3.3. BiLSTM Architecture

BiLSTM is a type of recurrent neural network, which is a class of neural network effective in time series regression tasks that temporally propagates information with each new estimate. As opposed to unidirectional LSTM models which only consider information from the past, BiLSTM models also consider information from future inputs to improve accuracy. The performance of BiLSTM was demonstrated in a similar study [18]. LSTMs mitigate the vanishing gradient problem prevalent in RNNs with a gated structure and cell state within each node. The BiLSTM used in this study was composed of two LSTM layers of size 50 and a fully connected layer that reshaped the network output to one size [28].

#### 2.3.4. BioMAT Architecture

Transformer models operate based on an attention mechanism. The original motivation behind developing transformer models was to solve natural transduction or language translation problems [30]. This model is ideal for sequence-to-sequence mapping [33]. Given the current study is related to mapping a sequence of IMU data to a sequence of joint kinematics, as well as the reliable performance of transformers in applications such as forecasting, transformer models are an ideal candidate.

**Figure 1:**
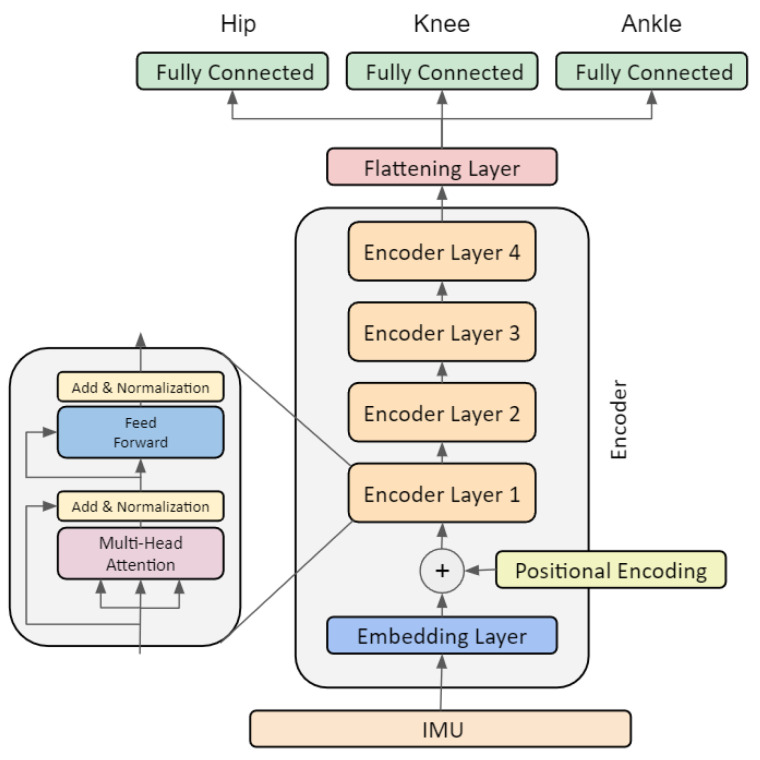
BioMAT’s architecture.

Transformer models can consist of an encoder and a decoder, which are connected by an attention layer. The encoder maps the input sequence to a vector representation. The decoder generates the output sequence from that vector representation. Bidirectional encoder representations from transformers (BERT) and generative pre-trained transformers (GPT) are two well-known systems that have been trained on large databases. BERT only includes an encoder and is typically trained using supervised learning for tasks such as text classification or named entity recognition [41]. GPT includes decoder and is trained using unsupervised learning. During training, the model learns to predict the next word or number in a sequence based on the previous context [30,42]. BioMAT was based on the BERT architecture with an encoder consisting of an embedding layer, a positional layer, and a stack of encoder layers each with multi-head attention layers followed by a flattening layer (Figure 1) [41]. Three additional fully connected layers were added to map the resultant vector from the encoder to three kinematics times series (hip, knee, and ankle). To prevent overfitting, dropout layers were added after positional encoding (res dropout) and after the flattening layer (fc dropout) (Table 1). The transformer model utilized in this study was adopted based on previously published work [43].

### 2.4. Training and Parameter Tuning

Data from the 19 subjects were randomly divided into training (16 subjects) and testing (3 subjects) sets. This subject allocation was selected to maximize the size of the training set while ensuring a minimum of three subjects in the test set to avoid the potential effects of outliers. Model training was conducted using adaptive learning rate optimization with a learning rate of 0.001, batch size of 50, and 50 epochs. The cost function used for training was the mean square error between predicted and measured kinematics. An L2 regularization coefficient of lambda = 0.001 was used to prevent overfitting. The models were created using PyTorch 1.8.1 in Python 3.7 and trained and evaluated on NVIDIA TiTAN XP GPUs with 12 GB of memory. Hyperparameters for the CNNLSTM and BiLSTM were implemented from the original studies [17,28]. Hyperparameters for BioMAT were selected after tuning with a 5-fold cross-validation by subjects on training data across all five activities. Table 1 includes the list of hyperparameters for each model.

### 2.5. Neural Network Evaluation and Statistical Tests

To investigate the generalizability of the models in predicting joint kinematics across various activities, the performance metrics of each model, after training on the combined dataset of all activities, were reported for predictions of each individual activity and predictions across all activities combined. The performance metrics included root mean square error (RMSE), normalized root mean square error (nRMSE), and Pearson correlation coefficient (r) between measured and predicted kinematics.

In a subsequent analysis, the models were re-trained for each activity separately (e.g., trained only on gait) and the predictions tested on that same activity as well as for activities not included in the training set (e.g., model trained on gait predicting stair ascent). The same evaluation metrics were used to assess the impact of activity diversity in the training datasets. Errors for tasks were aggregated by taking the mean across all joints of a specific activity and test subjects. A two-way multivariate analysis (MANOVA) was conducted to detect interactions between the two independent variables, training activities and test activities, and RMSE and r for BioMAT. A multiple comparison test was also conducted to compute pairwise differences between models trained on different training activities for each test activity. 

## 3. Results

**Figure 2:**
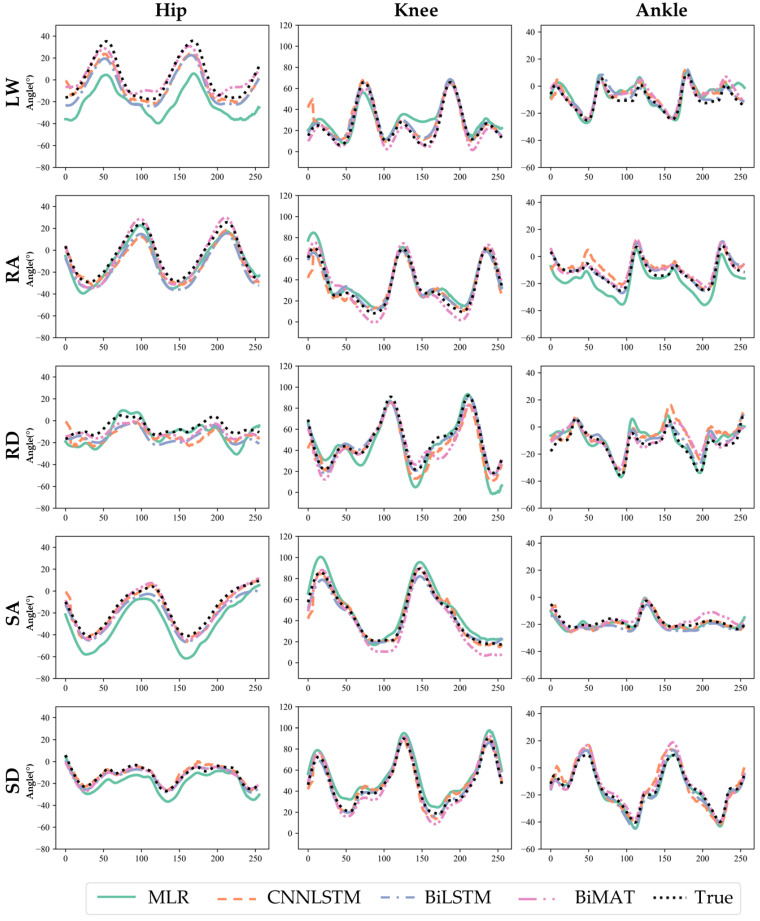
Representative ground truth and predicted joint kinematics across different activities and for a test subject from models trained simultaneously on all activities. Ground truth (dash line) and prediction (solid) for different predictive models (LW: Level Walking, RA: Ramp Ascent, RD: Ramp Descent, SA: Stair Ascent, SD: Stair Descent).

All machine learning models evaluated in the study produced joint kinematic predictions with higher accuracy than the baseline MLR for each activity after training simultaneously on all activities (Figure 2). BioMAT achieved lower RMSE across all three joints (5.5 ± 0.5°) compared to BiLSTM (7.0 ± 1.0°), CNNLSTM (8.8 ± 2.3°), and MLR models (14.1 ± 7.3°, Table 2). A similar trend was observed for nRMSE with the smallest nRMSE of 5.4 ± 1.2 for BioMAT and the largest nRMSE of 24.2 ± 12.7 for MLR. The mean correlation coefficients between model predictions and measured kinematics ranged from 0.91 ± 0.04 to 0.98 ± 0.01 for the MLR model at ankle joint and BioMAT at the knee, respectively. The BiLSTM model outperformed the other model architectures with the highest correlation coefficients at the knee (0.98 ± 0.02), hip (0.97 ± 0.04), and ankle (0.96 ± 0.02). BioMAT had equivalent performance with coefficients of 0.98 ± 0.01, 0.97 ± 0.04, and 0.95 ± 0.02 for the knee, hip, and ankle, respectively. Conversely, mean correlation coefficients were lower for the MLR (0.92 ± 0.04) and the CNNLSTM model (0.92 ± 0.05).

When trained on specific activities, BioMAT likewise demonstrated the lowest RMSE and nRMSE among model architectures for all five tasks with average RMSE and nRMSE across the three joints of 5.5 ± 1.1° and 6.8 ± 1.6°, respectively. BioMAT yielded similar correlation coefficients to BiLSTM for LW (0.97 ± 0.03), RA (0.97 ± 0.02), and SD (0.98 ± 0.02), and was slightly higher for RD (0.94 ± 0.02) and lower for SA (0.97 ± 0.04) (Table 3).

**Figure 3:**
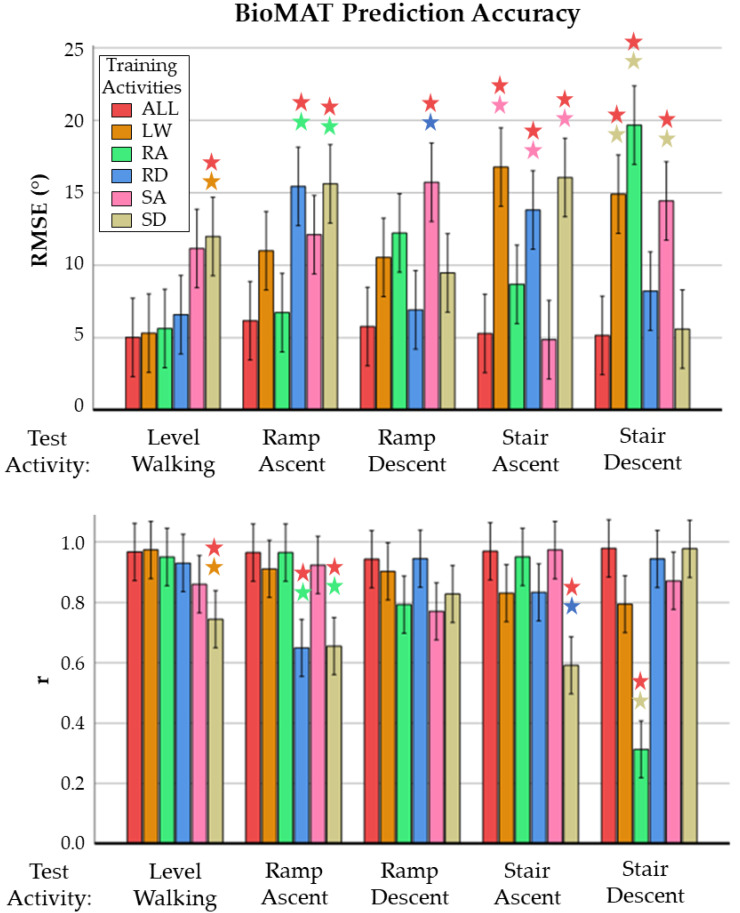
Mean RMSE (**top**) and r (**bottom**) of BioMAT’s kinematic predictions across different activities of daily living when trained on all activities versus individual activities. Stars indicate activities with statistically significantly worse predictions compared to models trained on other sets of activities (*p* < 0.05). (ALL: all activities, LW: Level Walking, RA: Ramp Ascent, RD: Ramp Descent, SA: Stair Ascent, SD: Stair Descent).

Increased activity diversity in the training set improved prediction accuracy for certain model architectures. For example, training the BioMAT and CNNLSTM architectures simultaneously on all activities improved prediction accuracy for four out of five activities compared to training on a specific activity (Table 3 and Table 4). However, the post hoc multiple comparison tests for BioMAT indicated the accuracy differences were not statistically significant. Conversely, training the MLR and BiLSTM architectures simultaneously on all activities reduced the prediction accuracy for three out of five activities. The two-way MANOVA identified significant main and interaction effects among the type of training data (all activities versus activity-specific) and test activity for both RMSE and r with BioMAT (F(50,142) = 2.674, *p* ≤ 0.001, Wilks’ Λ = 0.265). As expected, statistically significant reductions in accuracy (RMSE and r) were observed when the activity-specific models were used to predict kinematics from other activities (Figure 3).

## 4. Discussion

This study introduced an adaptation of a state-of-the-art transformer-based model (BioMAT) for predicting joint kinematics of lower extremities based on streams of IMU data including acceleration and angular velocity. BioMAT consistently yielded the highest performance with the lowest RMSE, nRMSE, and highest correlation coefficients compared to other published models at all three joints and across all five activities of daily living. When trained with data from all activities of daily living, BioMAT’s prediction accuracy was improved compared to training purely on activity-specific data for four out of five tasks. Further, this performance was achieved without the need to segment the input IMU data into discrete gait cycles, which stands as a key finding of our study. By eliminating the need for gait segmentation, this approach simplifies the preprocessing stage, reduces computational complexity, and potentially improves the real-time performance of gait analysis systems.

Zerveas et al. introduced a transformer-based model for multivariate time series representation learning in 2020 [33]. Their modeling approach generated the most accurate method for multivariate time series classification and regression tasks on several benchmark datasets when compared to contemporary models such as XGboost [44] and ResNet [45]. Siddhad et al. demonstrated that the transformer model outperformed BiLSTM and CNN models in a study to classify electroencephalograms [46]. These studies built the foundation for the current transformer-based model for multivariate time series in joint kinematic predictions from IMUs. BioMAT was likewise compared against CNNLSTM [17] and BiLSTM [28] architectures, the predominant models in recent literature for joint kinematic predictions, showing compelling results. The proposed BioMAT model demonstrated superior prediction accuracy with an average RMSE of 5.5° across all three joints and activities, compared to BiLSTM and CNNLSTM with average RMSEs of 7.0° and 8.8° respectively. BioMAT also achieved smaller standard deviations in RMSE across all joints and tasks (BioMAT standard deviations: joint level = 0.5° and task level = 1.1°), compared to BiLSTM and CNNLSTM (BiLSTM standard deviations: joint level = 1.0° and task level = 2.1°, and CNNLSTM standard deviations: joint level = 2.3° and task level = 3.7°). The smaller standard deviations demonstrate the increased reliability of the transformer compared to other models. 

The machine learning models used in this study improved the prediction accuracy relative to the benchmark MLR by 37% to 61% for RMSE, 69% to 93% for nRMSE, and up to 5% for correlation coefficients. The hip and ankle joints had the largest and smallest RMSE across all models, respectively. When normalized over the range of the kinematics data, the knee joint achieved the lowest nRMSE. This indicated that the deep learning models were most robust for the knee joint, then the ankle, and lastly the hip. The correlation coefficients were consistent across the joints for all models. The highest correlations were observed for the knee and the lowest for the ankle. Earlier studies have also observed this trend [28]. A plausible explanation for decreased predictive ability at the ankle joint is that the smaller range of motion generates a reduced signal-to-noise ratio in the IMU measurements [13,15,18,47]. In contrast, the hip joint’s larger range of motion did not necessarily result in better predictions due to increased soft tissue artifacts when tracking pelvis movements with both IMUs and the gold standard MOCAP. Previous studies have demonstrated lower accuracy in MOCAP measurements for the hip joint compared to the knee and ankle, which adversely affects the accuracy of the model predictions [48,49]. When comparing model performance across different tasks, BiLSTM and BioMAT had similar accuracy, with the lowest average RMSE of 5.3 ± 1.6° and 5.0 ± 1.5° for LW and the largest RMSE with values of 7.5 ± 2.1° and 6.2 ± 1.1° for RA, respectively. In contrast, the CNN model had its lowest accuracy for LW with an average RMSE of 12.3 ± 5.6°, and its best performance in SA with an RMSE of 6.8 ± 2.3°.

Mundt et al. evaluated an LSTM model’s kinematics predictions during level walking at different speeds using approximately 88,000 simulated IMU samples from 150 subjects and achieved an RMSE of 1.6°, 1.7°, and 1.4° with r of 0.98, 0.99, and 0.94 across the hip, knee, and ankle joints respectively [16]. Hernandez et al. employed a CNNLSTM model with 27 subjects performing treadmill walking and running tasks with reported mean absolute errors of 3.8°, 3.0°, 4.9°, and r of 0.99, 0.99, and 0.97 across three joints [17]. Compared to these studies, BioMAT had higher RMSEs (hip = 6.8°, knee = 4.2°, ankle = 4.2°), mean absolute error (hip = 5.5°, knee = 3.3°, ankle = 3.3°), and a lower correlation coefficient for the ankle joint (0.93). This was likely due to a combination of factors, including the larger training sets used in the previous studies, the use of simulated IMU data instead of measured IMU data, and performing walking on a treadmill instead of level ground. Simulated IMU data calculated from the kinematics of a musculoskeletal model do not include noise or skin artifacts inherent in measured IMU data. We have demonstrated in previous work that including synthetic IMU data improves prediction accuracy [18]. Treadmill walking also provides a more controlled environment compared to walking on level ground, resulting in more repeatable gait patterns [15,16]. Table 5 compares the current results to other similar studies that used measured IMU signals for model training. While BioMAT demonstrated comparable results, it should be noted that datasets, sensor positions, numbers of sensors, and environmental conditions varied between studies. Training models using a public dataset, such as the one used in this study, helps to standardize studies and can facilitate benchmarking various models and methodologies [15]. 

The accuracy of both BioMAT and the CNNLSTM models were statistically equivalent when trained simultaneously on all activities compared to task-specific models. This is likely because all activities considered in this study were variations of ambulation and had generally similar lower-limb motion patterns. The NN-models were robust enough to recognize subtle differences in IMU signals between similar activities such as walking down a ramp versus walking downstairs and predict the associated kinematics. This improves the generalizability of the tool and removes the need for activity classification and gait cycle segmentation prior to kinematic predictions. It remains unclear whether incorporating non-ambulatory activities (e.g., sit-to-stand) or ambulatory movements with significant out of plane movements (e.g., pivoting) would further improve the model’s generalizability and should be investigated in future work. In addition to the gains in prediction accuracy, BioMAT required less training and inference time for kinematic predictions (Table 6). Specifically, the inference time from BioMAT was 0.003 s/batch, 79% faster than predictions from the BiLSTM model [30,33]. The reduction in inference time has practical implications for applications that require real-time gait feedback, such as incorporation into control systems for active orthoses. Further, if deploying BioMAT on mobile devices with limited computational resources, the algorithm would employ less overall processor time.

There were multiple limitations to this study. First, the dataset used in this study included multiple configurations of stair height (four heights: 102 mm, 127 mm, 152 mm, and 178 mm) and ramp inclination angles (6 inclination angles of 5.2°, 7.8°, 9.2°, 11°, 12.4°, and 18°) as well as different speeds for level walking. Including greater variability in the training dataset likely improved the models’ generalizability, however, the effect of each configuration on the models’ performance was not examined and was outside the scope of the current study. Application of the current models to new datasets should be carried out with caution, as variations in sensor placement and sensor accuracy may adversely affect model predictions. Second, the sensitivity of each model’s performance to the number of data points in the inputs (e.g., 256) has not been investigated. Using a longer sequence length may further improve the models’ accuracy [16,18]. Third, the hyperparameters associated with the CNNLSTM and BiLSTM were selected based on previously reported studies to allow a direct comparison to the published results. It is plausible that hyperparameter tuning may improve the prediction accuracy of these models. Fourth, the current study focused on evaluating the performance of BioMAT for activity-specific and multi-activity training sets, but the contribution of each training activity to the final model performance remains unclear. Finally, the current study was limited to predicting joint angles in the sagittal plane and the accuracy of the proposed model for predicting joint angles in the coronal and axial planes has not been evaluated. Since the current model and dataset are both open-sourced, future researchers could leverage the current method and model to address some of these limitations.

In future work, the current model could be used to investigate machine learning techniques such as transfer learning [50,51], fine-tuning, and one- or zero-shot learning methods [52,53] for relevant biomechanical tasks or datasets. Transfer learning is a powerful technique to achieve highly accurate results on a wide range of tasks [54,55]. In biomechanics, kinematic prediction models trained on one dataset (e.g., gait activities in a healthy population) could be used with transfer learning to evaluate a new task or patient population (e.g., stair ascent in the OA population). Although BioMAT was trained on a healthy population, the tool could be fine-tuned to OA and total joint arthroplasty populations by adding a small number of observations from those groups. This would reduce the need to collect large quantities of data, preprocess samples, and train models from scratch, saving time, money, and computational resources [55]. Additionally, developing subject-specific models could improve the accuracy for temporal studies measuring changes in a subject’s movement patterns during interventional treatments such as rehabilitation. Synthetic data could potentially be used to overcome the hurdle of collecting large subject-specific training datasets, as we have demonstrated in a previous study [18]. Finally, knowledge distillation could potentially be used to compress the current model to a smaller model without significant loss in performance, improving computational efficiency for deployment on edge devices such as smartphones with limited hardware and resources [56].

## 5. Conclusions

In this study, we evaluated a deep learning transformer architecture, BioMAT, to estimate lower extremity kinematics from a continuous stream of IMU data for multiple activities of daily living. This model was trained using a publicly available dataset. BioMAT predicted joint kinematics with equivalent or lower errors than conventional deep NN models without the additional computational steps associated with activity classification and segmentation of gait cycles. This comprehensive analysis revealed that training the model on a diversity of activities outperformed models trained on specific activities in four out of five tasks. The primary scientific contribution of this research was demonstrating that a gait measurement equipped with a single multifunction transformer model relying on streams of unsegmented and unclassified IMU data can bridge the gap to real-time applications of wearable sensors for monitoring movement in clinical and commercial applications. 

## Figures and Tables

**Table 1:** Selected hyperparameters for each model.

CNNLSTM [18]	BiLSTM [27]	BioMAT
CNN2D-1 kernel size: 10, 3	BiLSTM hidden size: 128	BioMAT d model: 256
CNN2D-1 n output: 16	BiLSTM n layers: 2	BioMAT n heads: 16
CNN2D-2 kernel size: 10, 3	dropout: 0.2	BioMAT d ff: 128
CNN2D-2 n output: 32		BioMAT n layers: 4
LSTM hidden size: 128		res dropout: 0.5
LSTM n layers: 2		fc dropout: 0.5
dropout: 0.2		

BioMAT d model: Total dimension of the model (number of features created by the model); BioMAT n heads: Parallel attention heads; BioMAT d ff: The dimension of the feedforward network model; res dropout: Amount of residual dropout applied in the encoder; fc dropout: Dropout applied to the final fully connected layer.

**Table 2:** RMSE, nRMSE, and r (mean ± standard deviation) between model predictions and ground truth kinematics for models trained on all activities simultaneously across all subjects in the test set. Bold indicates most accurate model architecture for that joint metric.

Metrics	Joint	Hip	Knee	Ankle	Mean
RMSE (°)	MLR	20.3 ± 11.8	10.1 ± 1.9	11.9 ± 8.3	14.1 ± 7.3
	CNNLSTM	10.9 ± 2.2	10.5 ± 3.9	5.09 ± 0.8	8.8 ± 2.3
	BiLSTM	9.2 ± 1.4	6.9 ± 1.1	4.8 ± 0.8	7.0 ± 1.0
	BioMAT	**6.4 ± 1.0**	**5.5 ± 1.1**	**4.6 ± 0.7**	**5.5 ± 0.5**
nRMSE	MLR	24.2 ± 12.7	10.0 ± 2.3	17.3 ± 10.0	17.2 ± 7.8
	CNNLSTM	13.5 ± 3.5	10.6 ± 4.7	8.1 ± 2.7	10.7 ± 3.2
	BiLSTM	11.6 ± 3.4	6.8 ± 1.0	7.5 ± 1.8	8.6 ± 1.0
	BioMAT	**7.9 ± 1.6**	**5.4 ± 1.2**	**7.1 ± 0.9**	**6.8 ± 0.3**
r	MLR	0.92 ± 0.06	0.95 ± 0.04	0.91 ± 0.04	0.92 ± 0.04
	CNNLSTM	0.92 ± 0.04	0.93 ± 0.06	0.91 ± 0.07	0.92 ± 0.05
	BiLSTM	**0.97 ± 0.04**	0.98 ± 0.02	**0.96 ± 0.02**	**0.97 ± 0.02**
	BioMAT	**0.97 ± 0.03**	**0.98 ± 0.01**	0.95 ± 0.02	0.96 ± 0.01

**Table 3:** RMSE, nRMSE, and r (mean ± standard deviation) between model predictions and ground truth kinematics for models trained on all activities and tested on individual activities. Bold indicates most accurate model Confarchitecture for that activity.

Metric	Model	Train: All Test: LW	Train: All Test: RA	Train: All Test: RD	Train: All Test: SA	Train: All Test: SD
RMSE°	MLR	8.5 ± 2.1	21.7 ± 10.3	22.5 ± 10.8	8.9 ± 3.4	9.0 ± 3.5
	CNNLSTM	12.3 ± 5.6	9.7 ± 3.8	7.8 ± 2.4	6.8 ± 2.3	7.5 ± 2.7
	BiLSTM	5.3 ± 1.6	7.5 ± 2.1	7.4 ± 2.1	7.5 ± 2.6	7.3 ± 2.8
	BioMAT	**5.0 ± 1.5**	**6.2 ± 1.1**	**5.8 ± 1.1**	**5.3 ± 1.6**	**5.2 ± 0.7**
nRMSE	MLR	11.8 ± 1.7	23.3 ± 9.4	27.7 ± 15.9	10.9 ± 3.0	12.3 ± 8.2
	CNNLSTM	16.3 ± 3.3	10.1 ± 2.1	9.2 ± 2.9	8.3 ± 1.6	9.8 ± 5.7
	BiLSTM	7.3 ± 1.7	8.0 ± 0.4	8.7 ± 2.8	9.4 ± 2.8	9.7 ± 6.3
	BioMAT	**7.2 ± 2.4**	**6.7 ± 0.4**	**6.7 ± 0.3**	**6.6 ± 1.4**	**6.8 ± 3.0**
r	MLR	0.92 ± 0.03	0.92 ± 0.04	0.87 ± 0.05	0.96 ± 0.04	0.95 ± 0.05
	CNNLSTM	0.85 ± 0.04	0.9 ± 0.04	0.92 ± 0.02	0.97 ± 0.03	0.95 ± 0.03
	BiLSTM	**0.97 ± 0.02**	**0.97 ± 0.01**	0.93 ± 0.02	**0.98 ± 0.02**	**0.98 ± 0.01**
	BioMAT	0.97 ± 0.03	0.97 ± 0.02	**0.94 ± 0.02**	0.97 ± 0.04	0.98 ± 0.02

LW: Level Walking, RA: Ramp Ascent, RD: Ramp Descent, SA: Stair Ascent, SD: Stair Descent.

**Table 4:** RMSE, nRMSE, and r (mean ± standard deviation) between model predictions and ground truth kinematics for models trained on a single activity and tested on that same activity. Bold indicates most accurate model architecture for that activity.

Metric	Model	Train: LW Test: LW	Train: RA Test: RA	Train: RD Test: RD	Train: SA Test: SA	Train: SD Test: SD
RMSE°	MLR	9.6 ± 3.5°	31.2 ± 10.6°	13.8 ± 2.4°	7.9 ± 3.5°	7.9 ± 1.3°
	CNNLSTM	6.2 ± 2.2°	10.3 ± 4.5°	8.3 ± 1.4°	13.4 ± 5.2°	18.8 ± 6.8°
	BiLSTM	5.5 ± 1.6°	8.2 ± 2.9°	7.0 ± 2.0°	5.3 ± 1.7°	7.2 ± 2.1°
	BioMAT	**5.3 ± 2.1°**	**6.7 ± 2.0°**	**6.9 ± 2.2°**	**4.9 ± 1.4°**	**5.6 ± 1.3°**
nRMSE	MLR	13.1 ± 3.2	33.2 ± 6.7	16.2 ± 2.2	9.5 ± 2.7	10.1 ± 3.6
	CNNLSTM	8.4 ± 1.6	10.6 ± 2.3	9.7 ± 1.2	16.0 ± 1.5	22.4 ± 0.9
	BiLSTM	7.5 ± 0.3	8.6 ± 1.2	**8.2 ± 2.4**	6.5 ± 1.2	9.5 ± 5.1
	BioMAT	**7.3 ± 2.3**	**7.1 ± 0.6**	8.2 ± 3.1	**5.9 ± 0.7**	**7.5 ± 3.9**
r	MLR	0.91 ± 0.04	0.90 ± 0.03	0.83 ± 0.06	0.96 ± 0.04	0.95 ± 0.02
	CNNLSTM	0.94 ± 0.03	0.91 ± 0.04	0.88 ± 0.03	0.70 ± 0.30	−0.02 ± 0.04
	BiLSTM	0.97 ± 0.03	0.96 ± 0.01	0.93 ± 0.03	0.98 ± 0.03	0.98 ± 0.01
	BioMAT	**0.97 ± 0.02**	**0.97 ± 0.01**	**0.95 ± 0.03**	**0.97 ± 0.03**	**0.98 ± 0.02**

LW: Level Walking, RA: Ramp Ascent, RD: Ramp Descent, SA: Stair Ascent, SD: Stair Descent.

**Table 5:** Prediction accuracies from previous studies for sagittal lower limb kinematics. Sensor locations included the pelvis (P), thigh (T), shank (S), and foot (F). Activities include level walking (LW), level running (LR), treadmill running (TR), ramp ascent (RA), ramp descent (RD), stair ascent (SA), and stair descent (SD).

				RMSE°			r		
Study	Activity	Model	Sensors	Hip	Knee	Ankle	Hip	Knee	Ankle
Dorschkey et al. [13]	LW + LR	2DCNN	PTSF	5.4	5.2	5.5	0.97	0.99	0.96
Gholami et al. [47]	TR	1DCNN	F	5.6	6.5	4.7	0.84	0.93	0.78
Tan et al. [28]	LW	BiLSTM	TS	NA	8.4	NA	NA	0.85	NA
Tan et al. [28]	SA	BiLSTM	TS	NA	9.7	NA	NA	0.95	NA
Tan et al. [28]	SD	BiLSTM	TS	NA	10.0	NA	NA	0.86	NA
Sharifi et al. [18]	LW	BiLSTM	PTSF	7.2	2.9	NA	0.88	0.99	NA
Hossain et al. [16]	LW	DeepBBWAVE-Net	FF	4.3	4.3	3.1	0.97	0.99	0.95
Hossain et al. [16]	RA	DeepBBWAVE-Net	FF	5.7	5.0	3.5	0.98	0.98	0.96
Hossain et al. [15]	RD	DeepBBWAVE-Net	FF	4.3	6.1	3.7	0.93	0.97	0.94
Hossain et al. [15]	SA	DeepBBWAVE-Net	FF	6.0	5.9	4.0	0.98	0.99	0.96
Hossain et al. [15]	SD	DeepBBWAVE-Net	FF	5.3	6.8	5.0	0.93	0.97	0.98
Current	LW	BioMAT	TSF	6.8	4.2	4.2	0.99	0.99	0.93
Current	RA	BioMAT	TSF	7.3	6.2	5.1	0.98	0.97	0.95
Current	RD	BioMAT	TSF	4.9	7.0	5.5	0.92	0.97	0.94
Current	SA	BioMAT	TSF	6.9	5.3	3.7	0.99	0.99	0.93
Current	SD	BioMAT	TSF	6.0	4.8	4.7	0.96	0.99	0.98

**Table 6:** Number of parameters, training time, and inference time for each model.

Model	# Parameters	Training Time (s/epoch)	Inference Time (s/batch)
BiLSTM	106,635,584	14.2	0.014
CNNLSTM	1,201,046	15.9	0.006
BioMAT	51,257,603	12.9	0.003

## Data Availability

BioMAT has been made open-source and can be found at the link below on 1 July 2023. Publicly available datasets and models offer valuable resources for other researchers to accelerate advancements in the biomechanics field. BioMAT is available at: https://digitalcommons.du.edu/biomat/.
